# Genetic Predisposition to Hepatocellular Carcinoma

**DOI:** 10.3390/metabo13010035

**Published:** 2022-12-25

**Authors:** Rosa M. Pascale, Diego F. Calvisi, Francesco Feo, Maria M. Simile

**Affiliations:** Department of Medical and Surgical Sciences and Pharmacy, University of Sassari, Via P. Manzella 4, 07100 Sassari, Italy

**Keywords:** hepatocellular carcinoma, F344 rats, BN rats, DUSP1, FOXM1, GNMT, *CYP1A*, *PARP1 NFKB* and *PREX2* genes, MYBL2 transcription factor

## Abstract

Liver preneoplastic and neoplastic lesions of the genetically susceptible F344 and resistant BN rats cluster, respectively, with human HCC with better (HCCB) and poorer prognosis (HCCP); therefore, they represent a valid model to study the molecular alterations determining the genetic predisposition to HCC and the response to therapy. The ubiquitin-mediated proteolysis of ERK-inhibitor DUSP1, which characterizes HCC progression, favors the unrestrained ERK activity. DUSP1 represents a valuable prognostic marker, and ERK, CKS1, or SKP2 are potential therapeutic targets for human HCC. In DN (dysplastic nodule) and HCC of F344 rats and human HCCP, DUSP1 downregulation and ERK1/2 overexpression sustain SKP2-CKS1 activity through FOXM1, the expression of which is associated with a susceptible phenotype. SAM-methyl-transferase reactions and SAM/SAH ratio are regulated by GNMT. In addition, GNMT binds to CYP1A, PARP1, and NFKB and PREX2 gene promoters. MYBL2 upregulation deregulates cell cycle and induces the progression of premalignant and malignant liver. During HCC progression, the MYBL2 transcription factor positively correlates with cells proliferation and microvessel density, while it is negatively correlated to apoptosis. Hierarchical supervised analysis, regarding 6132 genes common to human and rat liver, showed a gene expression pattern common to normal liver of both strains and BN nodules, and a second pattern is observed in F344 nodules and HCC of both strains. Comparative genetics studies showed that DNs of BN rats cluster with human HCCB, while F344 DNs and HCCs cluster with HCCP.

## 1. Introduction

Hepatocellular carcinoma (HCC), a frequent human cancer, represents about 90% of primary liver cancers and is the second leading cause of cancer-related deaths yearly. Among the main risk factors of HCC development are infection with hepatitis B and C viruses, alcohol consumption, and aflatoxin B1 ingestion [1]. The development of HCC occurs through intermediate steps involving inflammation or cirrhosis, induced by different mechanisms, including absence of telomerase reverse transcriptase [2], and mutations of *CTNNB1*, *ARID2*, *ARID1A*, *TSC1/TSC2*, *RPS6KA3*, *KEAP1*, *MLL2* [3], and *TP53* genes [3,4]. Hepatitis C virus (HCV) is a leading etiology of HCC. The interaction of HCV with the human host is complex, in part depending on the fact that HCV is an RNA virus that cannot be integrated in the host’s genome. The mechanisms of HCV-induced HCC include activation of multiple host pathways as liver fibrogenic and survival pathways, and interaction with the immune and metabolic systems.

Most liver preneoplastic lesions re-differentiate or do not further evolve to cancer, whereas the presence of predisposing factors can influence the course of the disease in a high proliferative class of patients: Hemochromatosis, Wilson disease, Tyrosinemia, alpha1-antitrypsin and glycogen storage diseases, HBC and HCV prevalence, and alcohol exposure, which are associated with poorer differentiation and high chromosomal instability. In the presence of predisposing factors, a subset of lesions acquires the capacity for autonomous growth and progresses to neoplastic nodules (dysplastic nodules, adenomas) and HCCs [5,6,7]. Furthermore, several genes involved in the regulation of growth and multiplicity of preneoplastic and neoplastic liver lesions influence genetic predisposition to HCC [8,9,10]. The effect of overexpression of immune checkpoint molecules, as programmed death-1 (PD-1), cytotoxic T-lymphocyte antigen 4 (CTLA-4), lymphocyte activating gene 3 protein (LAG-3), and mucin domain molecule 3 (TIM-3), on tumor and immune cells and the high levels of immunosuppressive cytokines inducing T cell inhibition, may be an important mechanism of HCC immune escape [11]. Therefore, the immunotherapy based on immune checkpoint inhibitors, eventually in combination with kinase inhibitors, anti-angiogenic drugs, chemotherapeutic agents, and locoregional therapies, could represent promised targets in the treatment of HCC [11,12]. This review summarizes the recent clinical studies, as well as ongoing and upcoming trials. Hydrodynamic injection, associated to the Sleeping Beauty transposon system or the CRISPR/Cas9 genome editing tool, allows a rapid and cost-favorable production of a variety of HCC models, characterized by the activation of some oncogenes and/or inactivation of tumor suppressor genes [13,14]. This procedure offers a flexible tool to study an in vivo preclinical murine model, and biochemical cross talks among multiple pathways, and to verify the drug-therapeutic potential against hepatocellular carcinoma during the hepatocarcinogenic process [14]. Initiated hepatocytes, developing after N-nitrosodiethylamine (DENA) injection, show a high growth rate characterizing the genetic susceptibility to murine hepatocarcinogenesis [13,15]. It has been found that multiple loci affect the genetic predisposition to hepatocarcinogenesis in mice [13]. Interestingly, mice and rat strains with variable predisposition to HCC show different behaviors of preneoplastic and neoplastic lesions when compared accordingly to functional genetic studies. The resistant strains cluster with human HCC with better prognosis (HCCB), in contrast to the lesions developing in mouse and rat susceptible strains, which cluster with human HCC with poorer prognosis (HCCP) [14,15,16]. HCC susceptibility controls the expression of c-*Myc*, a gene that plays a central role in malignant conversion during human hepatocarcinogenesis [15,16,17]. The object of the present review is to offer a deeper knowledge on the molecular alterations involved in the achievement of a resistant or susceptible phenotype to hepatocarcinogenesis, in order to favor the approach to HCC therapy.

## 2. Molecular Mechanisms Determining the Susceptibility to Hepatocarcinogenesis

The study on HCC development in mouse and rat strains, which are differently susceptible to hepatocarcinogenesis, indicates the presence of a deregulation of G1 and S phases in the development of HCC in the genetically susceptible F344 rats and a G1-S block in lesions of the resistant Brown Norway (BN) rats [18]. Twelve weeks after initiation, preneoplastic lesions are more numerous but smaller in BN resistant than in F344 rats. Dysplastic nodules (adenomas) and poorly differentiated HCCs are present at 32 and 57 weeks, respectively, only in F344 rats. Moderately differentiated carcinomas develop in about the 70% of F344 rats. In BN rats, almost all liver nodules are constituted by clear/eosinophilic cells at 32 weeks from chemical carcinogen initiation, without atypical features. HCC lesions develop at 60 weeks in F344 rats [18]. It has been shown, by comparative study of the molecular pathways involved in mice and rat strains with different susceptibility to HCC: genes responsible for HCC susceptibility control the amplification and/or the overexpression of c-Myc, the expression of cell cycle regulatory genes, the activity of Ras/Erk and AKT/mTOR cascades, the pro-apoptotic properties of the Rassf1A/Nore1A and Dab2IP/Ask1 axes, the methionine cycle, and DNA repair pathways [17,18].

**Figure 1:**
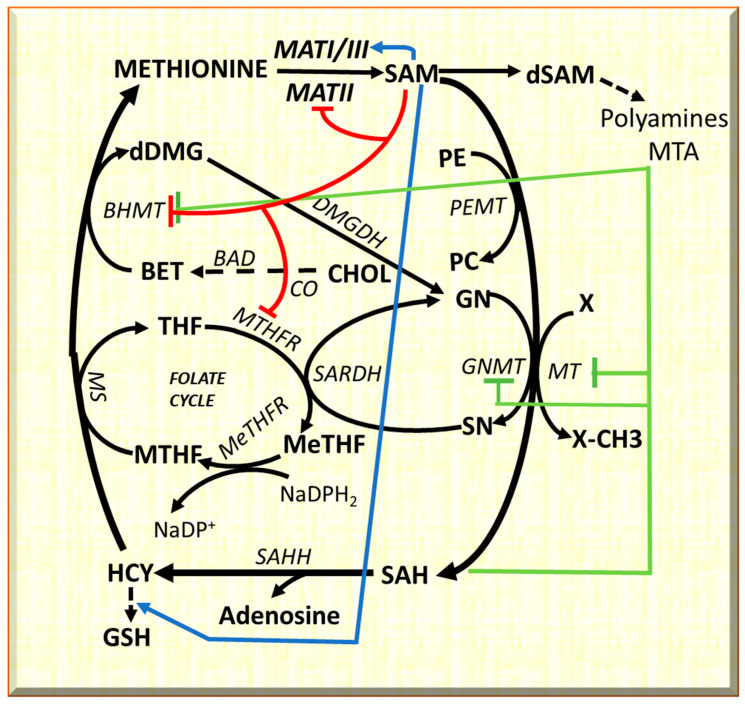
Metabolic cycles implicated in methionine metabolism, and long-range interactions of SAM. The inhibitory effects are indicated in red for SAM; and in green for SAH. The activator effects of SAM are shown in blue. Substrates: BET, betaine; CHOL, choline; DMG, dimethylglycine; dSAM, S-adenosylmethionine decarboxylated; GN, glycine; GSH, reduced glutathione; HCY, homocysteine; MTHF, methyltetrahydrofolate; MeTHF, 5,10-methenyl-tetrahydrofolate; PC, phosphatidylcholine; PE, phosphatidylethanolamine; SAH, S-adenosylhomocysteine; SAM S-adenosylmethionine; SN, sarcosine; THF, tetrahydrofolate. Enzymes: BAD, betaine aldehyde dehydrogenase; BHMT, betaine homocysteine-methyltransferase; CO, choline oxidase; DMGDH, dimethylglycine dehydrogenase; GNMT, glycine N-methyltransferase; MATI/III, MATII, methyladenosyltransferases I/III and II; MS, Methionine synthetase; MT, methyltransferases; MTHFR, methyltetrahydrofolate reductase; MeTHFR, 5,10-methenyl-tetrahydrofolate reductase; SAHH S-adenosylhomocysteine hydroxylase; SARDH, sarcosine dehydrogenase.

Evident differences between F344 and BN rats, susceptible or resistant to chemically induced hepatocellular carcinoma, respectively, concern the methionine cycle. In mammals, the methionine cycle, trans-sulfuration pathway, and polyamine biosynthesis require methionine [19] (Figure 1), and the last leads to SAM by methyladenosyltransferase I/III (MATI/III) and methyladenosyltransferase II (MATII). Methylation reactions, catalyzed by different methyltransferases, lead to S-adenosyl-homocysteine (SAH). SAH hydroxylase transforms SAH into homocysteine (HCY). Homocysteine may be transformed to cystathionine, a precursor of reduced glutathione, or may be used for methionine re-synthesis. Betaine synthesis, following the transformation of phosphatidylethanolamine to phosphatidylcholine by phosphatidylethanolamine methyltransferase (PEMT), or the folate cycle allowing tetrahydrofolate (THF) transformation to 5,10-methylenetetrahydrofolate (MeTHF), a reaction catalyzed by methyltetrahydrofolate reductase, are associated to the re-synthesis of glycine from sarcosine (Figure 1). MTHF is converted to THF by methionine synthetase, and the recovered methyl group is used to convert homocysteine into methionine (Figure 1). SAM is also a precursor of polyamines and 5-methylthioadenosine (MTA). A specific nucleosidase transforms MTA to methylthioribose, further available for methionine resynthesis in the so-called “salvage pathway”.

The methionine and folate cycles interact with cell metabolism [19] (Figure 1). SAM “long-range interactions” include GSH synthesis from HCY, and BHMT [20,21,22] and MTHFR inhibition [23,24] with consequent restraint of methionine resynthesis and purine and pyrimidine synthesis. Furthermore, GNMT regulates the SAM/SAH ratio and SAM-dependent methyl-transfer reactions. GNMT Km for SAM is relatively high, and SAH does not inhibit GNMT because its Ki value for SAH (35–80 μM) is higher than that for other SAM-dependent methyltransferases that are highly inhibited by SAH [22]. Therefore, GNMT is active at SAM and SAH physiological levels (0.1–0.2 μmol/g and 0.02–0.06 μmol/of the liver, respectively) and may influence the activity of other methyltransferases. GNMT protein binds folate and is inhibited by MTHF [20,21]. Therefore, the latter’s concentration is reduced due to the inhibition of MTHFR by SAM. This event induces the dissociation of GNMT-MTHF complex [23,24,25,26], and the increasing level of free GNMT avoids excessive SAM amount. On the contrary, if SAM concentration decreases, MTHFR inhibition is released, MTHF availability increases, and the free GNMT falls [27,28,29].

## 3. The Role of Genetic Mechanisms Regulating the Susceptibility to HCC

**Figure 2:**
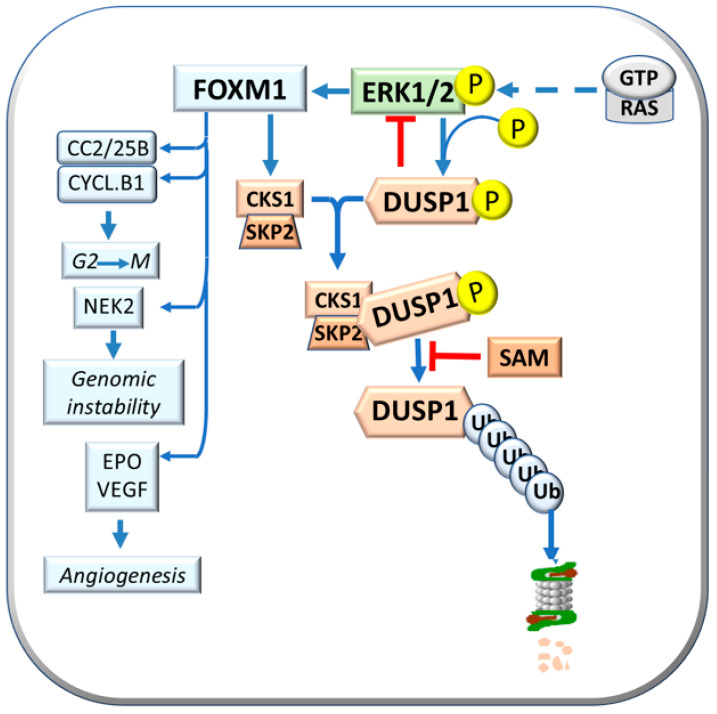
Effects of FOXM1: activation of CKS1 and SKP2 expression and CKS1/SKP2 complex formation, also known as “Ubiquitin ligase”, with consequent phosphorylated DUSP1 proteasomal degradation, and activation of Cdc2/25B/Cyclin B1, NEK2, and EPO.

Previous work in our laboratory showed a faster progression to HCC of initiated cells in F344 rats, genetically susceptible to hepatocarcinogenesis, and a slower progression in the resistant BN rats. In this strain, initiated cells evolve slowly, most preneoplastic lesions re-differentiate, and only few progress to HCCs [29]. Supervised hierarchical analysis of 6132 genes common to rat and human liver showed that dysplastic nodules (DNs) and HCCs of the resistant BN rats cluster with human HCC with better prognosis (HCCB), while most DNs and all HCCs of the susceptible F344 rats cluster with human HCC with a poorer prognosis (HCCP) [30]. SAM may control the MAPK (mitogen-activated protein Kinase) pathway. Indeed, SAM may induce a decrease in ERK1/2 activity by stimulating the expression of DUSP1, a specific ERK inhibitor (Figure 2). DUSP1 downregulation and ERK1/2 overexpression characterize fast progressing DN and HCC of F344 rats and human HCCP [30,31]. On the other hand, active ERK1/2 phosphorylates the Ser296 residue of DUSP1, causing its ubiquitination by the CKS1-SKP2 ubiquitin ligase and proteasomal degradation [31,32]. ERK1/2 sustains CKS1-SKP2activity through its target FOXM1 (forkhead box M1) [33] (Figure 2). Notably, DUSP1 mRNA and protein levels decrease in MAT1A-KO mouse livers and cultured mouse and human hepatocytes [32]. SAM treatment protects against the decrease of DUSP1 in cultured mouse and human hepatocytes. In MAT1A-KO mice, the increase in SAM and Dusp1 mRNA and protein levels is associated with a reduction of Erk1/2 activity [32]. ERK achieves unrestrained activity during HCC progression by determining the ubiquitin-mediated proteolysis of its specific inhibitor, DUSP1. Therefore, DUSP1 represents a valuable prognostic marker, and ERK, CKS1, or SKP2 are potential therapeutic targets for human HCC [32].

FOXM1 upregulation is due to the combined activity of extracellular ERK and glioblastoma-associated oncogene 1 (GLI1). Once activated, FOXM1 overexpression increases proliferation and angiogenesis and reduces apoptosis in human HCC cell lines [34]. FOXM1 is associated with acquiring a susceptible phenotype in rats and influences human HCC development and prognosis [34,35,36]. Furthermore, FOXM1 induces the transcription of Cyclin B1, Cdc2, and Cdc25b (cell division cycle 25B) that regulate G2-M transition; *NEK2* (never in mitosis gene kinase 2) gene, involved in genomic instability; and antiapoptotic and angiogenesis genes such as *SURVIVIN*, *EPO* (Erythropoietin), and *VEGF* [36] (Figure 2). ERK1/2 upregulation is associated with low DUSP1 expression in fast-growing DNs and HCCs induced in F344 rats, genetically susceptible to hepatocarcinogenesis, and human HCCs with poorer prognosis (based on patient’s survival length) [35].

**Figure 3:**
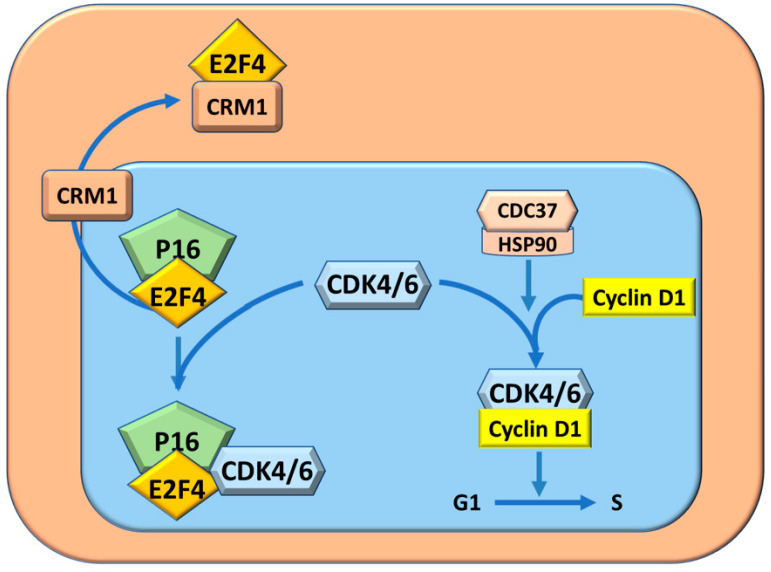
Cell cycle protection from inhibition by P16INK4A through the complex CDC37/HSP90 and CRM1. The complex P16/E4 by sequestering CDK4/6, impedes the formation of the complex CDK4/6-CyclinD2 and consequently inhibits cell cycle.

The molecular mechanisms determining the resistant phenotype of BN rats have been analyzed. Preneoplastic liver (7 wks. after initiation), neoplastic nodules (32 wks.), and HCC (54–56 weeks) show a lower level of p16INK4A mRNA and protein in F344 than in BN rats [37]. This is associated with an increase of the expression of Heat shock protein 90 (Hsp90) and its complex formation with CRM1 transporter protein. The latter transports E2F4 outside the nucleus (Figure 3), thus inactivating p16INK4A. In addition, the HSP90-Cell division cycle 37 (Cdc37) complex protects Cdk4 and Cdk6 kinases from the formation of inhibitory complexes with p16INK4A/E2F4 [37,38,39] (Figure 3). The increase in the Cdc37-Cdk4 complex activates cyclin D1 and the cell cycle, and decreases the p16INK4A-Cdk4 complex in the lesions of susceptible F344 rats, whereas lower/no changes occur in BN rats [38,39,40].

**Figure 4:**
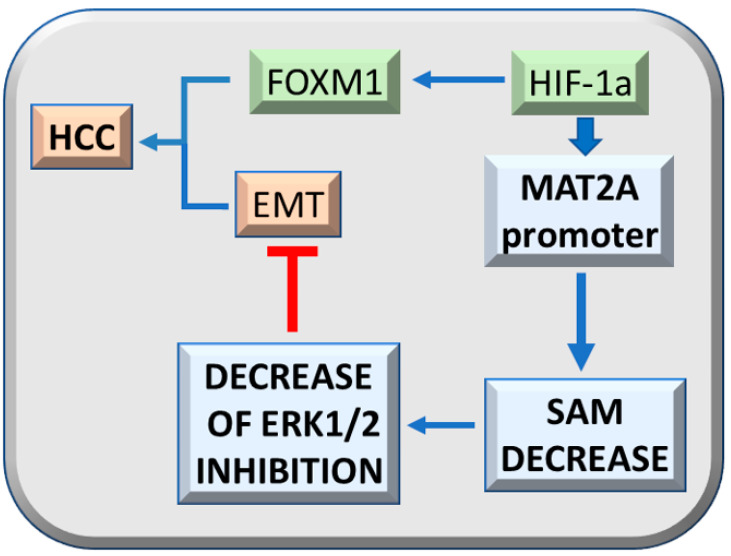
Effect of HIF-1a on hepatocarcinogenesis. The HIF-1a axis sustains, together with TNF-a, FOXM1 expression and tumorigenicity. HIF-1a binding to MAT2A promoter inhibits SAM production and the consequent ERK1/2 inhibition by SAM, while it favors epithelial/mesenchymal transition (EMT) and tumorigenesis.

Interestingly, HIF-1a and the TNF-a/HIF-1a axis sustain the expression of FOXM1 [41,42] that mediates ERK1/2 effects on the cell cycle, cell survival, and angiogenesis [33]. Hypoxia contributes to ERK1/2 upregulation by decreasing the SAM content of HCC cells through HIF-1a binding to the MAT2A promoter [43] (Figure 4). The consequent lower inhibition of ERK1/2 by SAM, favors the ERK1/2 regulated epithelial/mesenchymal transition (EMT) [44]. These observations support a suppressive effect of SAM on malignant transformation through ERK1/2 inhibition by multiple mechanisms, such as genetic predisposition, including increased transcription and stability of its mRNA and protein, and inhibition of proteasomal chymotrypsin-like and caspase-like activities [45].

## 4. GNMT and the Genetic Predisposition to HCC

**Figure 5:**
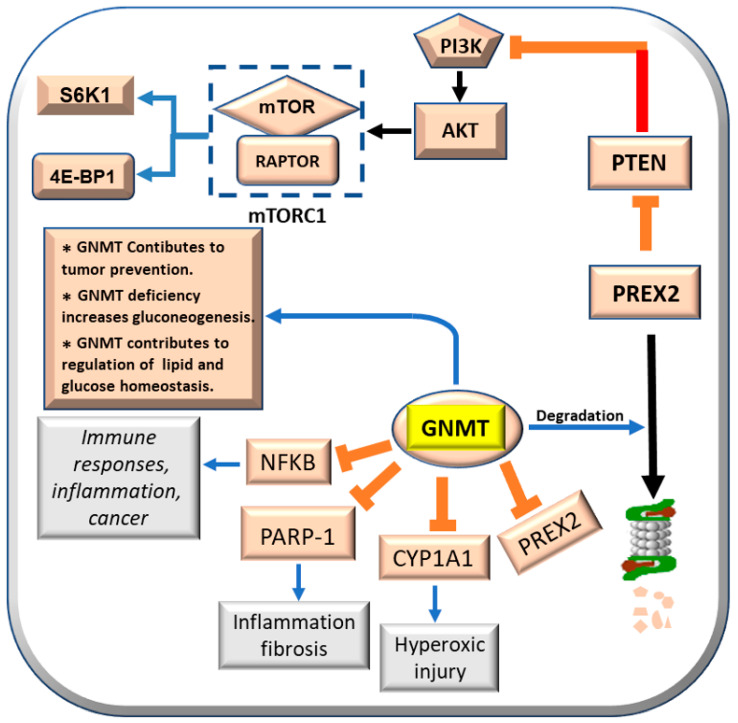
Effects of GNMT on CYP1A1, PARP-1, NFKB, and PRERX2, and its contribution to tumor prevention, gluconeogenesis, lipid, and glucose homeostasis.

GNMT exerts, at molecular level, a multifaceted suppressive action by interacting with various cancer-related genes and inhibiting their expression. GNMT nuclear localization suppresses hepatocarcinogenesis [44]. Indeed, GNMT overexpression binds to the promoters of *CYP1A1* (Cytochrome p450-1A1) [46], *PARP1* [Poly (ADP-ribose) polymerase 1] [46,47], *NFKB* (nuclear factor-kB) [48], and *PREX2* [49], and inhibits their expression (Figure 5). As a consequence, GNMT inhibits bronchopulmonary dysplasia that follows oxidative stress [50,51], inflammation and fibrosis induced by PARP1 [47], and the role of NFKB in inflammation, immune response, and cancer [52]. Further, GNMT induces the proteasomal degradation of PREX2 [50]. This impedes PTEN inhibition by PREX2 [49,53]. Consequently, PTEN (phosphatase and tensin homolog deleted on chromosome 10) inhibits the PI3K/AKT/mTORC1/S6K1/4E-BP1 pathway [53] (Figure 5). However, GNMT may also have some beneficial effects. Gnmt knockout mice develop liver injury, fibrosis, and HCC [54]. GNMT-/- female mice develop lipid and glucose homeostasis impairment following increased Akt (pAkt) signaling [55,56]. Aflatoxin B1 metabolism and its carcinogenic activity effect is modulated by GNMT [44,57]. Nuclear localization of the GNMT protein was found to be higher in human HCC with better prognosis (HCCB) than in HCC with poorer prognosis (HCCP) [45]. GNMT forced expression inhibits proliferation, induces apoptosis, and suppresses GNMT *CYP1A1*, *PREX2*, *PARP1*, and *NFKB* gene expression by binding to their promoters in vitro in Huh7 and HepG2 cell lines [45]. This observation indicates that GNMT protein exerts a polyhedric suppressive activity during hepatocarcinogenesis, interacting and inhibiting the expression of several cancer related genes.

**Figure 6:**
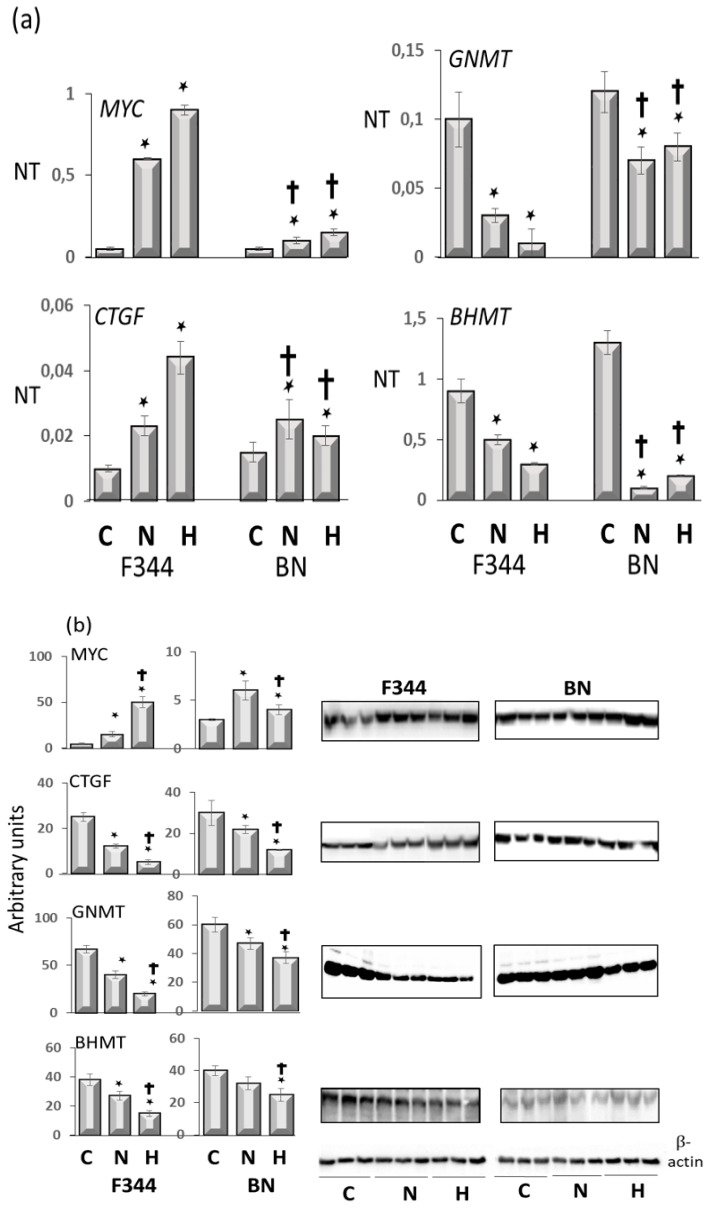
(**a**): qPCR analysis of Myc, Ctgf, Gnmt, and Bhmt RNA expression in normal liver (C), dysplastic nodules (N), and HCC (H) of F344 and BN rats. Results are means ± SD of six normal livers, 15 DNs 32 weeks after initiation, and 14 HCCs per strain. Number Target (NT). NT = 2-ΔCt, ΔCt = CT (target)–CT(RNR-18). Tukey–Kramer test: N and H vs. C, BN vs. F344, at least *p* < 0.05 for all genes tested. (**b**): Western blot of c-Myc, Ctgf, Gnmt, and Bhmt proteins expression. Optical densities of the peaks were normalized to β-actin values and expressed as arbitrary units. Data are means ± SD of three normal livers, five DNs, and five moderately-differentiated HCCs from F344 and BN rats. Tukey–Kramer test: (*) N and H vs. C, at least *p* < 0.05. (†) F344 vs. BN, *p* < 0.001.

Furthermore, to investigate the relationship between the oncogenes *Myc* and *Ctgf* and the oncosuppressors *Gnmt* and *Bhmt* with the genetic predisposition to HCC, the levels of these genes were determined in preneoplastic liver lesions and HCCs from F344 rats, genetically susceptible to hepatocarcinogenesis, and in those resistant BN rats. The analysis revealed two distinctive gene expression patterns, the highest expression of c-*Myc* and *Ctgf*, and the lowest expression of oncosuppressors *Gnmt* and *Bhmt* in more aggressive DNs and HCCs of susceptible F344 rats (Figure 6a,b, R.M. Pascale et Al. unpublished results). Therefore, these observations strongly suggest that the GNMT oncosuppressive activity influences several genes implicated in hepatocarcinogenesis.

## 5. MYBL2 Gene and Liver Cancer

**Figure 7:**
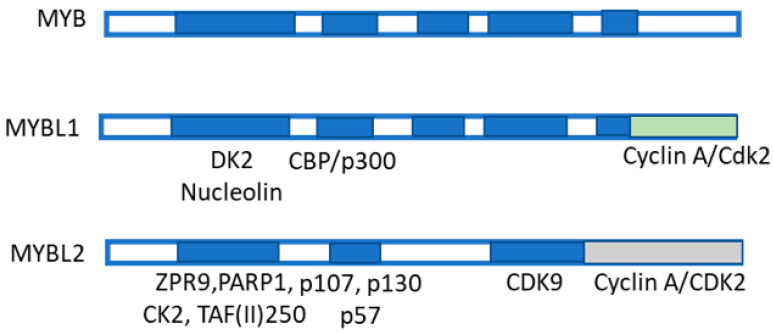
Structure of MYB family members and interacting proteins. MYB family members are shown: they include MYBL1 and MYBL2. MYB-binding domain, made up of the three repeats R1, R2, and R3 binding to several proteins, among which include p100, PARP, Cyp40, c-Ski, N-CoR, C/EMPbeta, RAR, SMRT, and mSin3A. The MYBL1 and MBL2 protein structures are also shown. MYB co-activators are listed in green. MYB co-repressors are also listed.

MYB transcription factors are highly conserved from plants to vertebrates, indicating their critical role in cell biology. In humans, the *MYB* gene family comprises three members: *MYB*, *MYBL1*, and *MYBL2*, encoding the transcription factors MYB, MYBL1, and MYBL2 (Figure 7), respectively [58,59]. Mutations and rearrangements of these genes are involved in cancer. Interestingly, c-MYBL2, like other transactivating factors such as Sp1, NF-kB, and AP-1, transcriptionally induces MAT2A in HCC [60]. Furthermore, the knockdown of OPA3 (Optic atrophy 3), a MYBL2 target overexpressed and associated with an unfavorable prognosis of HCC patients, reduces glucose uptake, glycolysis and ATP production, and HCC cell growth [59]. Furthermore, MYBL2 knockdown decreases HCC aerobic glycolysis while OPA3 overexpression reverses these alterations [59]. These findings indicate that the MYBL2-OPA3 axis enhances HCC aerobic glycolysis and proliferation. In addition, c-MYB and Sp1 are overexpressed in HCC, and the mutation of the c-MYB or Sp1 site reduces MAT2A promoter activity [58,61]. Thus, c-MYB and Sp1 overexpression and binding to the MAT2A promoter contribute to MAT2A transcriptional up-regulation in HCC [61].

Proliferation, genomic instability, and microvessel density are positively correlated to MYBL2 expression during HCC progression, while they negatively correlate with programmed cell death (apoptosis). The upregulation of *MYBL2* gene, observed in faster growing preneoplastic and neoplastic rat liver lesions, and in human HCC suggest that Mybl2 expression is under genetic control, and contributes to cell cycle and signal transduction deregulation. Interestingly, human HCCP show lower retinoblastoma protein (pRB) activation, and the highest level of *MYBL2*, and of E2F1-DP1 complex and activated Cyclin B1, if compared to human HCCB [61]. In conclusion, a lower overall survival is associated to the highest MYBL2 expression and MYBL2-increased transcription regulatory activity in patients bearing primary HCC with poorer prognosis [62,63].

These results disclose a significant role of oncosuppressor genes as effectors of genetic resistance to hepatocarcinogenesis (Table 1). Furthermore, comparative functional genomic analyses showed the existence of an evolutionarily conserved gene expression signature that allows the recognition of HCC phenotypes with a different propensity to progress to more malignant lesions in rats and humans. Our observations suggest that the rat hepatocarcinogenesis model enables the identification of prognostic subgroups of human HCC and new putative prognostic markers [62,63].

## 6. HCC Modifiers and Genetic Predisposition

**Figure 8:**
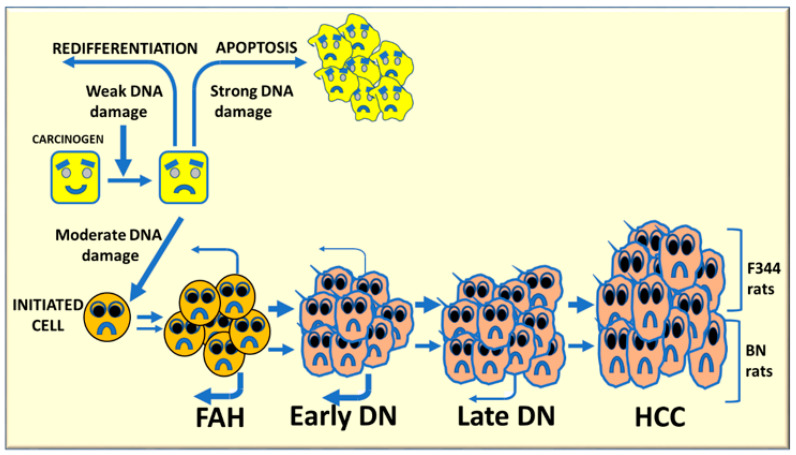
Schematic representation of multistage hepatocarcinogenesis. Most initiated cells undergo death by apoptosis or re-differentiate to normal appearing phenotype, depending on the gravity of the carcinogenic stimulus. A strong stimulus irreversibly affects DNA resulting in cell death, whereas a weak stimulus can allow DNA repair and redifferentiation (retroverted arrows). Intermediate stimuli could be compatible with cell survival, and possible cell transformation. On the other hand, moderate DNA damage may induce carcinogenesis initiation. Several rounds of cell division, during the selective expansion of initiated cells, trigger the development of foci of altered hepatocytes (FAH), early dysplastic nodules (DNs), late DNs, and hepatocellular carcinomas (HCC).

The resistant phenotype of various mouse and rat strains depends on the incapacity of preneoplastic cells to grow autonomously and evolve into cancer, as well as on a high re-differentiation rate [64,65,66,67]. Most initiated cells die by apoptosis during multistage hepatocarcinogenesis or re-differentiate into a normal-appearing phenotype (Figure 8). These events could at least partially depend on the severity of the carcinogenic stimulus [66]; strong stimuli may irreversibly affect DNA and DNA repair mechanisms, thus resulting in cell death. In contrast, weak stimuli could allow (and trigger) DNA repair and re-differentiation. Increased cytoplasmic expression of p53, Bcl-2, and NFKB occurs in persistent preneoplastic lesions [68].

The role of re-differentiation in the acquisition of the resistant phenotype has been shown in the Copenaghen (Cop) rat strain, resistant to hepatocarcinogenesis. In these rats, the re-differentiation is responsible of the block of progression of preneoplastic lesions, although DNA synthesis in these lesions proceeds at the same rate as in the lesions of susceptible rats [65,66]. Phenotypic reversion can implicate re-differentiation and/or apoptosis of initiated hepatocytes [67]. The analysis of BN and Cop rats excluded apoptosis as a mechanism of resistance to hepatocarcinogenesis and attributed a preeminent role to the re-differentiation [64,68]. While autonomous growth of preneoplastic lesions was very small or absent in the resistant strains, many persistent nodules evolve to HCC in the susceptible F344 rats, [8,69].

The results of the study on the effects of QTLs (quantitative trait loci) on phenotype and some molecular pathways of rat strains with different sensitivity to liver cancer, indicate that several QTLs prevent genomic instability of initiated cells and favor their re-differentiation [8,16]. This behavior is compatible with a polygenic predisposition to HCC, the functional connection of multiple genes related to hepatocarcinogenesis, with minor to moderate effects on quantitative traits, allows a significant quantitative impact.

Hepatocellular carcinoma is a complex polygenic disease [70], and the BN rat strain carries dominant hepatocarcinogen resistance loci [71]. Initiated cells may have a different destiny during multistage hepatocarcinogenesis (Figure 8). A weak carcinogenic stimulus can allow DNA repair and cell re-differentiation, whereas a strong impulse may irreversibly affect DNA repair mechanisms, resulting in cell death. Intermediate stimuli may cause genomic instability with upregulation of oncogenes and growth factors and down-regulation of oncosuppressor genes, and initiation of some hepatocytes. Several rounds of cell division, during the selective expansion of initiated cells, lead to the development of foci of altered hepatocytes (FAH), early nodules larger than a liver lobule, and then late dysplastic nodules (adenomas) and carcinomas. This process proceeds faster in F344 rats, genetically susceptible to hepatocarcinogenesis, in which re-differentiation (remodeling) of FAH is low and further decreases in early nodules. FAH and early DN strongly re-differentiate in resistant BN rats, and a low-grade of re-differentiation still occurs in late DN in these rats [68,71,72].

The unsupervised hierarchical analysis of 6,132 genes common to rat and human liver [72,73] showed two different gene expression patterns: the first involved normal liver of both strains and BN nodules, and the second one involved F344 nodules and HCC of both strains. Notably, a signature predicting DN and HCC progression, characterized by the overexpression of *Csmd1*, *Dmbt1*, *Dusp1*, and *Gnmt* oncosuppressor genes in DNs, and *Bhmt*, *Dmbt1*, *Dusp1*, *Gadd45g*, *Gnmt*, *Napsa*, *Pp2ca*, and *Ptpn13* genes in HCCs of resistant rats, was identified. Integrated gene expression data showed the highest expression of proliferation-related *CTGF*, *c-MYC*, and *PCNA* genes and the lowest expression of *BHMT*, *DMBT1*, *DUSP1*, *GADD45g*, and *GNMT* genes in more aggressive rat and human HCC. *BHMT*, *DUSP1*, and *GADD45g* expression predicted patients’ survival. These results revealed, for the first time, a crucial role of oncosuppressor genes in determining the genetic resistance to hepatocarcinogenesis. Furthermore, comparative functional genomic analyses discovered an evolutionarily well conserved gene expression signature that could distinguish HCCs prognosis [73]. The unsupervised hierarchical cluster analysis of gene expression patterns of rat liver tissues revealed that in DN and HCC, the features of 1362 gene showed more than a 2-fold difference compared to the median expression values.

**Figure 9:**
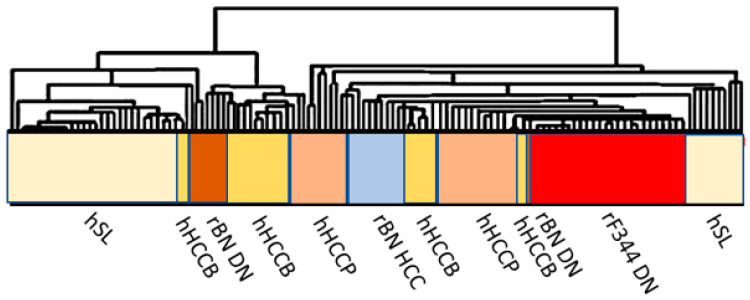
Comparative functional genomics approach by integrated unsupervised hierarchical cluster analysis of 28 human surrounding non-tumorous livers, 35 HCCB, 35 HCCP, and rat surrounding liver, DN and HCC. Abbreviations: hSL and rSL, human and rat surrounding liver, hHCCB, human HCC with better prognosis; rBN DN, BN rat dysplastic nodules; rF344 DN, F344 rats dysplastic nodules; rBN HCC, BN rats hepatocellular carcinomas; hHCCP, human hepatocellular carcinomas with poorer prognosis; rF344 HCC, F344 rats hepatocellular carcinomas.

The study of rodent models clearly showed a polygenic predisposition of HCC development determining the cancer phenotype, and involved one or few highly penetrant cancer-related genes and several modifier genes acting by multiple epistatic interactions. Similar model could be applied to human hepatocarcinogenesis, accordingly to population research. Comparative functional genetics studies identified the best-fit of mouse [74] and rat [75,76,77] models of hepatocarcinogenesis. The supervised hierarchical analysis of the 6,132 genes, common to rat and human liver, showed that DNs and HCCs of BN rats, and F344 DNs cluster with human HCCB, and F344 DNs and HCCs cluster with HCCP (Figure 9). These observations confirm the strength of the studies in mouse and rat models on the influence of genetic predisposition to hepatocarcinogenesis and on HCC prognosis.

## 7. Conclusions

Mutations of the genes involved in hemochromatosis, alpha 1-antitrypsin deficiency (*SERPINA1*), glycogen storage diseases (*G6PC*, *SLC37A4*), porphyries (*HMBS*, *UROD*), tyrosinemia (*FAH*), and Wilson’s Disease (ATP7B) increase the susceptibility to HCC [3,4,5,7,73]. Furthermore, several common conditions or diseases inherited as polygenic traits, i.e., autoimmune hepatitis, type 2 diabetes, a family history of HCC, hypothyroidism, and non-alcoholic steatohepatitis (NAFLD), also show an increased risk of HCC compared to the general population [72,73]. The results of recent research on the alterations of signaling transduction involved in hepatocarcinogenesis showed that different alterations, accounting for the acquisition of a susceptible phenotype during rat hepatocarcinogenesis, also contribute to human hepatocarcinogenesis; a major locus and multiple low-penetrance loci play a role in various circumstances [34,71]. Recent research on signaling transduction driving rat hepatocarcinogenesis showed that several alterations accounting for the acquisition of a susceptible phenotype, contribute to human hepatocarcinogenesis also, with a major locus and multiple low-penetrance loci playing a role during liver cancer development and progression [72,73,74,75]. Population studies have shown that a similar model applies to human hepatocarcinogenesis. Therefore, the knowledge of liver tumor epigenetics is fundamental for the diagnosis, prognosis, and therapy of human HCC. Several reports suggest the increasing severity of some human diseases, including HCC, following COVID-19 infection. It could be of interest the evaluation on the influence of COVID-19 pandemic on HCC development and progression [76,77]. Comparative functional genetics studies on the best-fit mouse [15,75] and rat [10,18] models of hepatocarcinogenesis showed that DNs and HCCs of BN rats, and F344 DNs cluster with human HCCB, while F344 DNs and HCCs cluster with HCCP. Furthermore, different genes contributing to the glycolytic metabolism of tumors, including, *RAS*, *MYC*, *LKB1*, *AMBK*, *HIF1*, are overexpressed in tumors [71,78,79,80,81]. Therefore, the studies on genetic predisposition to HCC have shown that a similar model applies to human hepatocarcinogenesis. These observations underline the utility of the studies on the influence of genetic predisposition to hepatocarcinogenesis.

## Figures and Tables

**Table 1:** Some important genes involved in HCC development.

GENES	ROLE IN HCC
MAT I/III (Adult liver)	Synthesis of SAM
MAT II (widely distributed)	Synthesis of SAM
PEMT/GNMT	Synthesis of SAH
SAHH	Synthesis of homocysteine
FOXM1	Contribution to DUSP1 proteasomal degradation
HIF-1 alpha	Activation of FOXM1
MYC, CTGF	Overexpressed in F-344 rat strain
PCNA	Overexpressed in F-344 rat strain
GNMT	Overexpressed in BN rat strain
BHMT	Overexpressed in BN rat strain
